# An improved preparation method for a CuO/CeO_2_-coated monolith for the CO–PrOx reaction

**DOI:** 10.1038/s41598-023-36423-7

**Published:** 2023-06-08

**Authors:** Jan Meißner, Lara Ahrens, Joachim Pasel, Alexander Schwedt, Sebastian Wohlrab, Joachim Mayer, Ralf Peters

**Affiliations:** 1 Institute of Electrochemical Process Engineering (IEK-14), Forschungszentrum Jülich, Jülich, Germany; 2 Central Facility for Electron Microscopy, RWTH Aachen University, Aachen, Germany; 3 Leibniz Institute for Catalysis Rostock, Rostock, Germany

**Keywords:** Chemistry, Energy science and technology

## Abstract

In this study, we present a method for directly coating monoliths with a CeO_2_/CuO catalyst using the urea-nitrate combustion method. The catalyst was characterized by means of XRD, SEM/EDX, and EPR measurements. Experimental results are described, when this catalyst was used for the preferential oxidation of CO. The catalytic activity for the CO–PrOx-reaction was measured by recording CO conversion as a function of the reaction temperature in a hydrogen-rich gas mixture in the presence and absence of water vapor. In a long-term test of over 310 h, the catalyst’s long-term stability was demonstrated. Direct coating is shown to be a promising approach by which a larger amount of catalyst can be deposited onto the monolith in a single step than would be possible with washcoats.

## Introduction

For the operation of a polymer electrolyte membrane fuel cell (PEFC), a hydrogen-rich fuel gas is generated from liquid hydrogen carrying energy vectors (e.g., diesel, kerosene or methanol) by the fuel processing technology. The first step of fuel processing is reforming, often followed by the water–gas shift reaction (WGS)^1^.

The latter has the function to decrease the concentration of CO in the product gas from reforming from approximately 8 to 10 vol.% to less than 1 vol.%. In addition, the product gas of a WGS reactor typically contains about 35 vol.% H_2_, 16 vol.% CO_2_, 20 vol.% H_2_O, 0.5 vol.% Ar, and a few hundred to a few thousand ppm methane, as well as other traces of higher hydrocarbons^2–5^, balance to 100% is nitrogen. However, for the operation of a PEFC, the CO concentration must be reduced further to values of < 100 ppm^1,6^. This is often performed by means of a CO–PrOx (preferential oxidation) reactor. It is planned to utilize a monolithic support coated with a catalyst free of noble metals in a future thermally integrated dual-stage PrOx-reactor.

For the choice of the catalyst system, the focus was on the technical process chain of a fuel processing system as described by Samsun et al.^4^. The CO-PrOx reactor must fit seamlessly into the system. The design temperature at the inlet of a PrOx reactor must therefore be based on the outlet temperature of the WGS reactor in the range of 200–300 °C. Based on a large number of promising published results^7–16^, the catalyst system CuO/CeO_2_ was chosen to fulfill this task. In the following, we report a targeted synthesis of this catalyst on a monolith support using the urea-nitrate combustion method.

## Experimental section

### Preparation of the monoliths

Based on the work of Avgouropoulos et al.^9^ in particular, as well as Barbatos et al.^11^ and Landi et al.^14,16^, ceramic monoliths made of cordierite with a honeycomb structure of 400 cpsi (Paul Rauschert GmbH & Co. KG, Germany) were coated with the mixed oxide CuO/CeO_2_. The monoliths were 70 mm long and 12 mm in diameter, resulting in a volume of 7.9 cm^3^.

However, no washcoat of the presynthesized oxide was used to coat the ceramic monoliths with the catalyst as had been done previously. Instead, the precursor solution for the CuO/CeO_2_ mixed oxide (Cu/(Cu + Ce) molar ratio of 0.15) documented by Avgouropoulos et al.^9^ was synthesized and the monoliths were dipped once (monolith #1) or twice (monolith #2) in this viscous solution. The monoliths coated with the precursor solution were then calcined at 450 °C, during which the catalyst formed directly on the monoliths surface. Compared to washcoats, the use of a viscous precursor solution offers the advantage of a more intimate adhesion to the support, while classical preparation methods, such as precipitation, are difficult to realize for obtaining homogeneous catalyst coatings. Additionally a powder was prepared from the precursor solution in the same manner for the purpose of materials characterization. After single coating and calcination, the applied amount of catalyst material was 446 mg in the case of monolith #1. In the case of monolith #2 a double coating was conducted, yielding a catalyst coating of about 768 mg.

### Characterization

#### XRD measurement

XRD measurement was performed on the powdered sample using a Bruker D8 Discover — Cu(Kα) = 1.5418 Å (tube voltage: 40 kV; current = 40 mA) without an Ni filter from 25° to 85°, with a step size of 0.02016° at 2 s/step.

#### SEM–EDX measurements

For EDX measurements, a cross-section of the monolith was polished with an ion cross-section polisher (JEOL, SM-09010) in order to obtain a plane surface. To increase the conductivity of the surface, gold was sputtered onto it. The microstructure and chemical composition of the catalyst were then analyzed by means of a scanning electron microscope (SEM) (Zeiss, Gemini SEM300) equipped with an EDX-detector (UltimMax 65, Oxford instruments). The images were acquired at 20 kV with a scan time of 2 ms/pixel. The EDX data were then processed by AZtec software (V. 5.1, Oxford Instruments).

#### Ex-situ EPR characterization

For the ex-situ EPR characterization, the spectra of the fresh monolith were recorded at −180 °C on a Bruker EMX CW-micro X-band spectrometer (ν ≈ 9.4 GHz) with a microwave power of 6.9 mW, a modulation frequency of 100 kHz, and an amplitude of 1 G. The spectrometer was equipped with a variable temperature control unit, including a liquid N_2_ cryostat and temperature controller. For the measurement, 54 mg of the as received sample was loaded in a quartz tube. The g values were calculated using the equation $h\nu =g\beta {B}_{0}$ with ${\beta , B}_{0}$, and $\nu$ being the Bohr magneton, resonance field, and frequency, respectively. Calibration of the g values was performed using a DPPH standard (g = 2.0036 ± 0.0004).

#### In-situ EPR measurements

For the in-situ EPR measurements, spectra were recorded in an ELEXSYS 500-10/12 X-band cw spectrometer (Bruker) with a modulation frequency and amplitude of 100 kHz and up to 5 G, respectively. The reaction was performed at 190 °C in a flow of 30 ml min^−1^ (40 vol.% H_2_, 2 vol.% O_2_, 1 vol.% CO, and balanced He) without any pre-treatment for the as received sample. Typically, 66 mg of the sample was loaded in a quartz plug-flow reactor connected to a gas dosing unit equipped with mass flow controllers (Bronkhorst) at the inlet, as well as a variable temperature control unit.

#### Activity measurements

For the experiments, the catalyst coated monoliths were wrapped with a ceramic fiber paper and inserted tightly into a double-walled steel cylinder. The monoliths were clamped in the tube by the ceramic fiber paper and thus held in the intended position. The temperature was measured at two points: First, directly after 5 mm in the direction of flow, and second, approximately 1 mm before the gas stream exited the monolith. The steel tube can be heated from the outside with a heating tape and, if necessary, cooled with an air flow in the annular gap in counter-flow in order to control the temperature at the inlet and the outlet of the monolith. The temperature between the inlet and outlet of the monolith was kept constant.

#### Instrumentation

Concentrations of CO and CO_2_ in the product gas were measured quasi-continuously at 10 s intervals using an FTIR (MKS Cirrus 2). Prior to measuring, the sample gas was dehumidified to a dew point of ≈ − 20 °C using a sample gas dryer (Perma Pure).

#### Experimental procedure

The educt gas mixtures were mixed from the pure gases and fully deionized water by means of mass flow controllers (MFCs), Bronkhorst El-Flow, and Liqui-Flow, respectively. Supplementary Figure SI1 shows a simplified R&I diagram of the test rig. Together with the gas stream of non-combustible gases as carrier gas, the demineralized water was evaporated in an evaporator unit (Bronkhorst CEM W-202A). Before entering the reactor, the three gas streams — non-combustible gases, combustible gases, and air — were mixed together. The piping downstream of the evaporator to the PrOx reactor and the piping from the reactor to the heat exchanger are heated to prevent re-condensation of steam.

In order to prevent condensation on the surface of the catalyst, the premixed gas mixtures were fed to the reactor at least 20 K above the dew point of the admixed water. The temperature in the reactor was initially continuously increased over a period of roughly two hours to about 250 °C in order to obtain an initial information on the catalyst’s performance. For this purpose, the CO concentration in the product gas was measured quasi-continuously every 10 s.

For a more accurate determination of the CO conversion, the temperature was subsequently increased and maintained in suitable increments from 80 °C beyond the point of maximum conversion until constant CO and CO_2_ concentrations could be measured in the product gas. The gas hourly space velocity (GHSV) was varied from GHSV = 5000 h^−1^ to 20,000 h^−1^ based on the monolith volume of 7.9 cm^3^ for the tested samples. This means the total volume flow rate was between about 40 Nl/h and 160 Nl/h.

#### Compositions of the utilized gas mixtures

- Fuel gas 1, dry (FG 1): 39 vol.% H_2_, 20 vol.% CO_2_, 1 vol.% CO, balance: N_2_.
- In its composition, fuel gas 1 approximates the fuel gas that a later CO-PrOx reactor is typically designed to convert, but is water-free.
- Fuel gas 2, moist (FG 2) 35 vol.% H_2_, 16 vol.% CO_2_, 1 vol.% CO, 20 vol.% H_2_O, balance: N_2_.
- Fuel gas 2 is a fuel gas that, except for a few hundred ppm of methane and other traces of hydrocarbons, corresponds to a typical fuel gas downstream of the water–gas shift reactor.

## Results and discussion

### Characterization

#### XRD measurements

**Figure 1:**
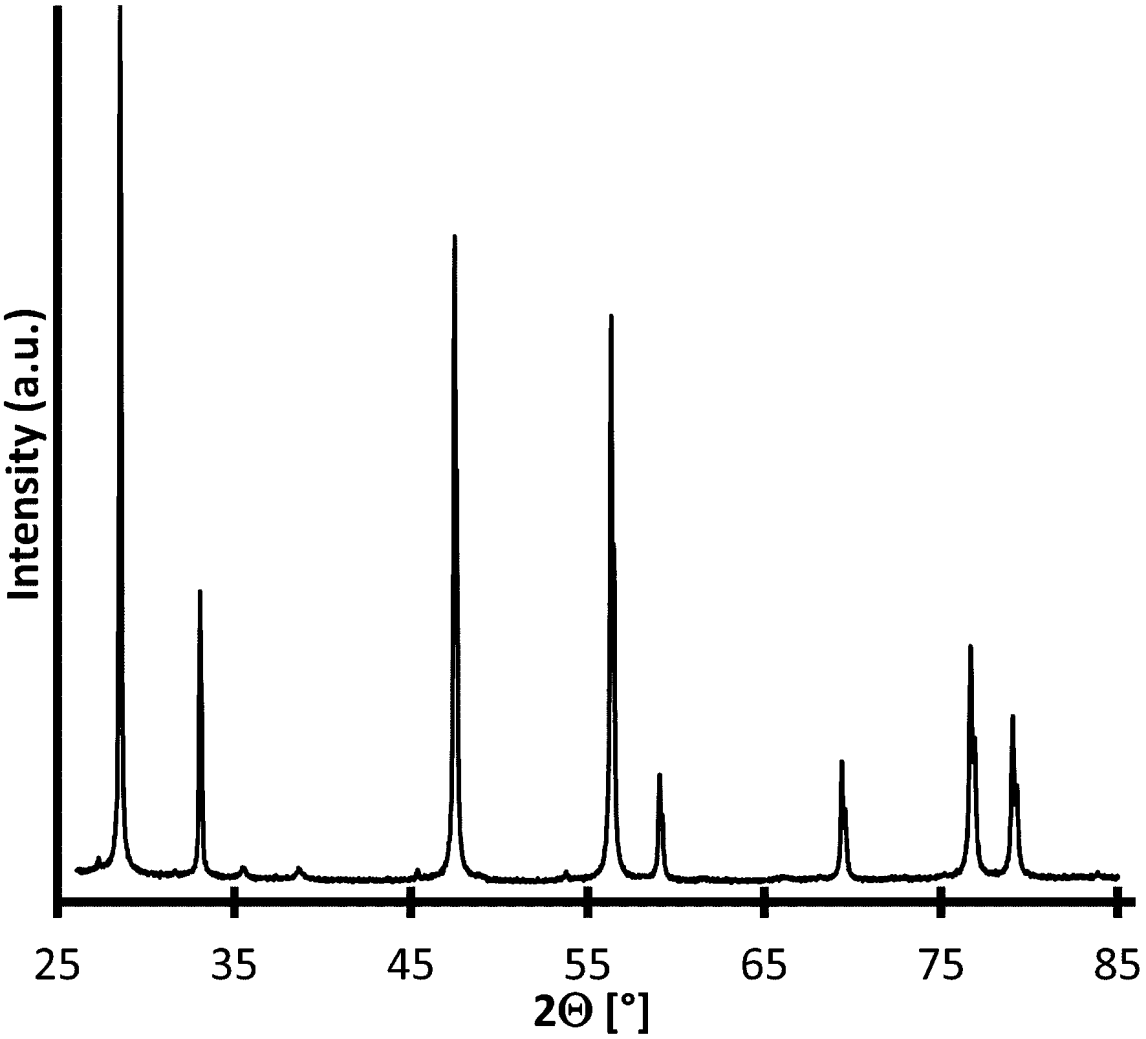
XRD diffractogram of powdered CuO/CeO_2_ catalyst.

The measured XRD diffractogram depicted in Fig. 1 is congruent with the pattern measured by Avgouropoulos et al.^9^. The signals show a high correlation with CeO_2_ in cubic form. However, CuO could not be clearly detected in the sample by means of XRD measurements, reflections of CuO with the highest intensity where to be expected at 2Θ angles of 35.5° and 38.8°. The exact state of CuO is discussed by Avgouropoulos et al.^9^. According to them, it is assumed to be either an amorphous phase or “highly dispersed clusters” of copper oxide on ceria^17–23^ or a solid solution where Cu^2+^ is integrated into the CeO_2_ lattice^24–26^.

#### SEM–EDX measurements

**Figure 2:**
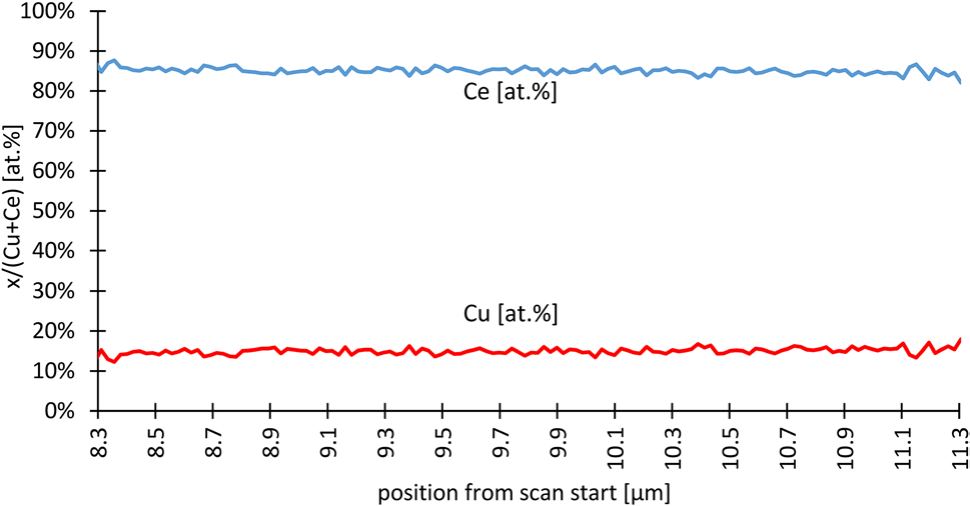
EDX line across the coating on monolith #1.

An EDX map was collected across a cross-section of the coating of monolith #1 on the wall of a central channel ≈ 5 mm from the entrance. From this, a layer-averaged line profile of the length of 16 µm was generated by averaging the spectra perpendicular to the line over a width of approximately 6 µm for each position starting in the wall of the monolith and ending in vacuum. An overview image of scan region is shown in Supplementary Fig. SI2. Here the Cu could be detected clearly in the catalyst layer on the monolith. The measurement (see Fig. 2) shows a high agreement for the Cu/(Cu + Ce) molar with the target value of 0.15. The averaged molar ratio for Cu/(Cu + Ce) over a distance of 3.0 µm in the catalytic layer was 0.1495 ± 9.7%, with a confidence interval of > 95%. Beyond the section shown in the figure, the conditions for a reliable quantification were not given even if the trend indicates a constant composition. Detailed data of the complete line scan can be found in the supplemental information.

**Figure 3:**
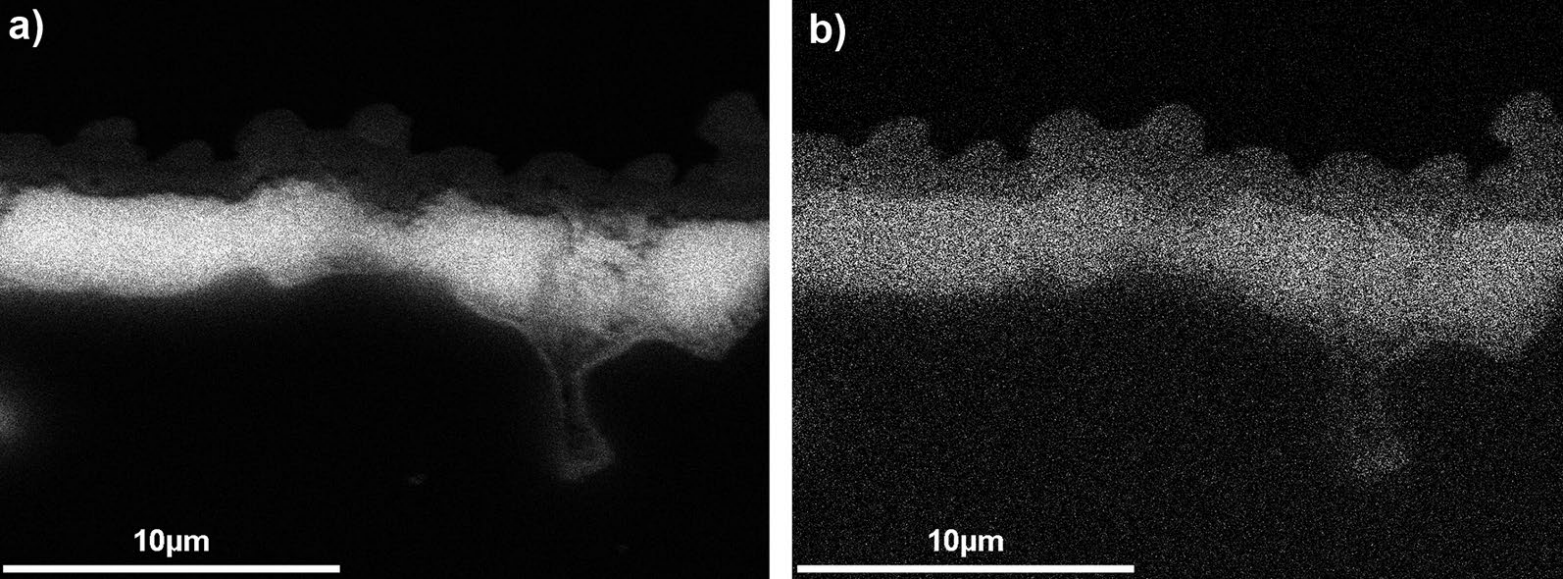
EDX mapping monolith #1, cross-section; (**a**) Ce; (**b**) Cu.

The homogeneous distribution of both elements could also be proven by SEM–EDX mappings. Figure 3 shows the homogeneous distribution of the smaller amount of copper (a) in a matrix of cerium oxide (b), analogous to the XRD data. In the upper part of the image, the rough catalyst surface can be seen, and at the bottom, the irregular surface of the cordierite support can be observed, as well as a pore in it on the right, which is partially filled with catalyst material.

This shows the strength of the synthesis method we have chosen: By applying a viscous precursor, a very intimate adhesion of the later catalyst material can be achieved, which even reaches structures that are very difficult to access, such as small pores. Compared to washcoat coatings^27^, a much more intimate adhesion of catalyst and carrier can be achieved.

#### Ex-situ EPR characterization results

**Figure 4:**
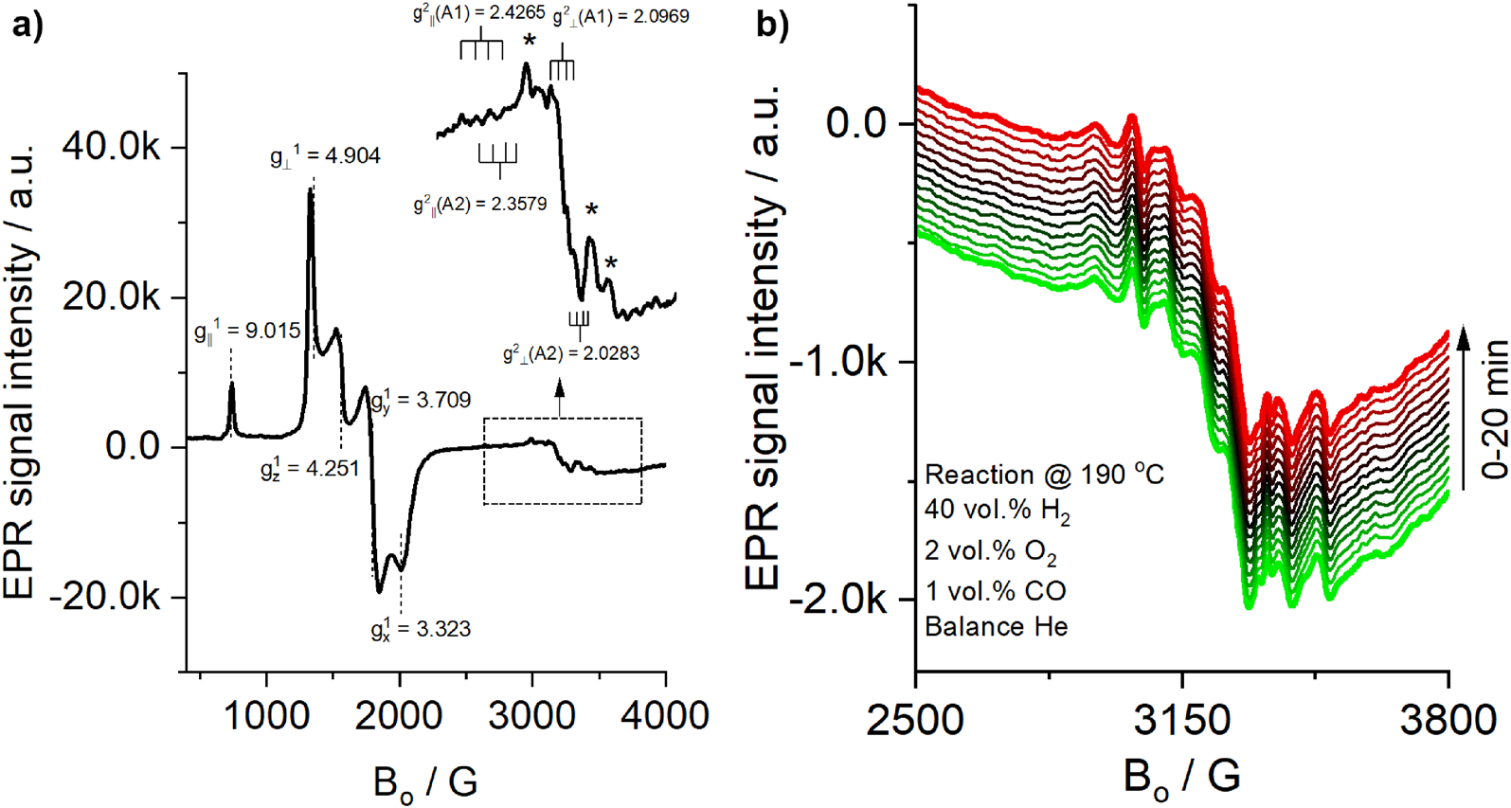
(**a**) EPR spectra of fresh monolith #1 recorded at – 180 °C. (**b**) EPR spectra recorded during reaction on the monolith #1 at 190 °C.

The EPR spectra of the monolith shown in Fig. 4a exhibited weak signals for two different isolated Cu^2+^ sites (shown in the inset) denoted as A1 species, which could be associated with tetragonally-distorted Cu^2+^ in the bulk of the support and A2 species, which might correspond to Cu^2+^ ions at surface substitutional sites with square-pyramidal symmetry^28–30^. It is worth noting that the existence of EPR-silent Cu^2+^ cannot be discarded, as strong magnetic interactions between antiferromagnetic coupling electrons of Cu^2+^ atoms in CuO might also be present^31–33^.

Additionally, typical signals of Fe^3+^ species with different environments and geometries appeared at g ~ 4.3 and g ≥ 6.0^34,35^. The signals at ${g}_{\| }^{1}=9.015$ and $g_{ \bot }^{1} = 4.904$ were assigned to a tetrahedrally-coordinated Fe^3+^ with an axial symmetry, whereas those at ${g}_{z}^{1}=4.251$, ${g}_{y}^{1}=3.709$ and ${g}_{x}^{1}=3.323$ were assigned to isolated Fe^3+^ with a rhombic symmetry.

#### In-situ EPR assessments

Figure 4b shows the spectra recorded in a time lapse of 20 min at 190 °C (40 vol.% H_2_, 2 vol.% O_2_, 1 vol.% CO, and balance He). The intensity of the EPR signals at the reaction temperature is weakened, and therefore the superimposition of Cu^2+^ signals with signals from Mn^2+^ makes it difficult to clearly observe the lines associated with the copper ions. Moreover, although it is well known that Cu sites might be active for the oxidation of CO due to the shuttling between Cu^2+^ and Cu^+^ with the participation of surface oxygen and oxygen vacancies^28,31,36^, it should be noted that during this time, no remarkable changes in the EPR spectra were detected. Hence, it is possible that the active Cu sites for the catalytic reaction are EPR-silent, as the catalytic test showed activity under similar conditions, in accordance with the ex-situ characterization results.

### Activity measurements

**Figure 5:**
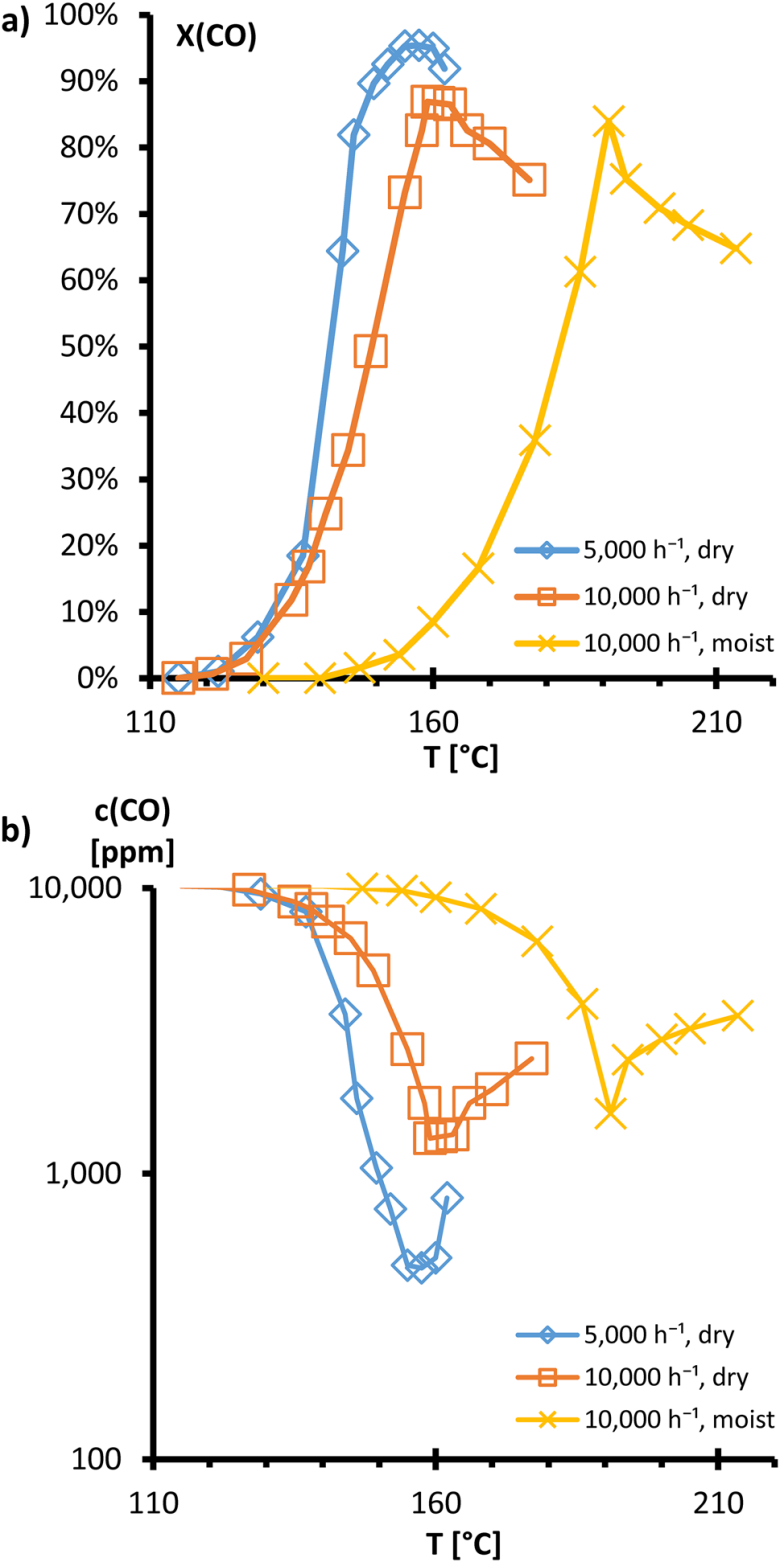
(**a**) CO conversion graphs, dry (FG1) and moist (FG2) fuel gas with single coated Monolith #1 (CuO/CeO_2_), all λ = 2.5, varying GHSV. (**b**) Corresponding CO concentrations at the outlet.

For single-coated monolith #1, with a CuO–CeO_2_ coating of 446 mg, CO conversions were measured as a function of the temperature and at different space velocities. In each case, the λ-value was $\uplambda =\frac{2 \dot{\mathrm{n}}({\mathrm{O}}_{2})}{\dot{\mathrm{n}}(\mathrm{CO})}$ = 2.5. The corresponding results are shown in Fig. 5. With the dry fuel gas (FG1) a conversion > 90% could be achieved at a GHSV of 5000 h^−1^. Between 155 °C and 160 °C, the CO conversion was higher than 95%. Increasing the space velocity GHSV to 10,000 h^−1^, a conversion exceeding 86% was achieved between 159 and 163 °C. The conversion curve for the moist fuel gas at a space velocity of 10,000 h^−1^ rises steeply up to a temperature of 191 °C, reaches X(CO) = 84% as a maximum, and then declines steeply again. The detected decrease of the CO conversion at higher reaction temperatures can be explained by the oxidation of H_2_, which becomes more and more dominating and consumes more and more oxygen. The observed shift of the temperature window is due to the shorter residence time of the molecules on the catalyst surface with increasing GHSV values.

**Figure 6:**
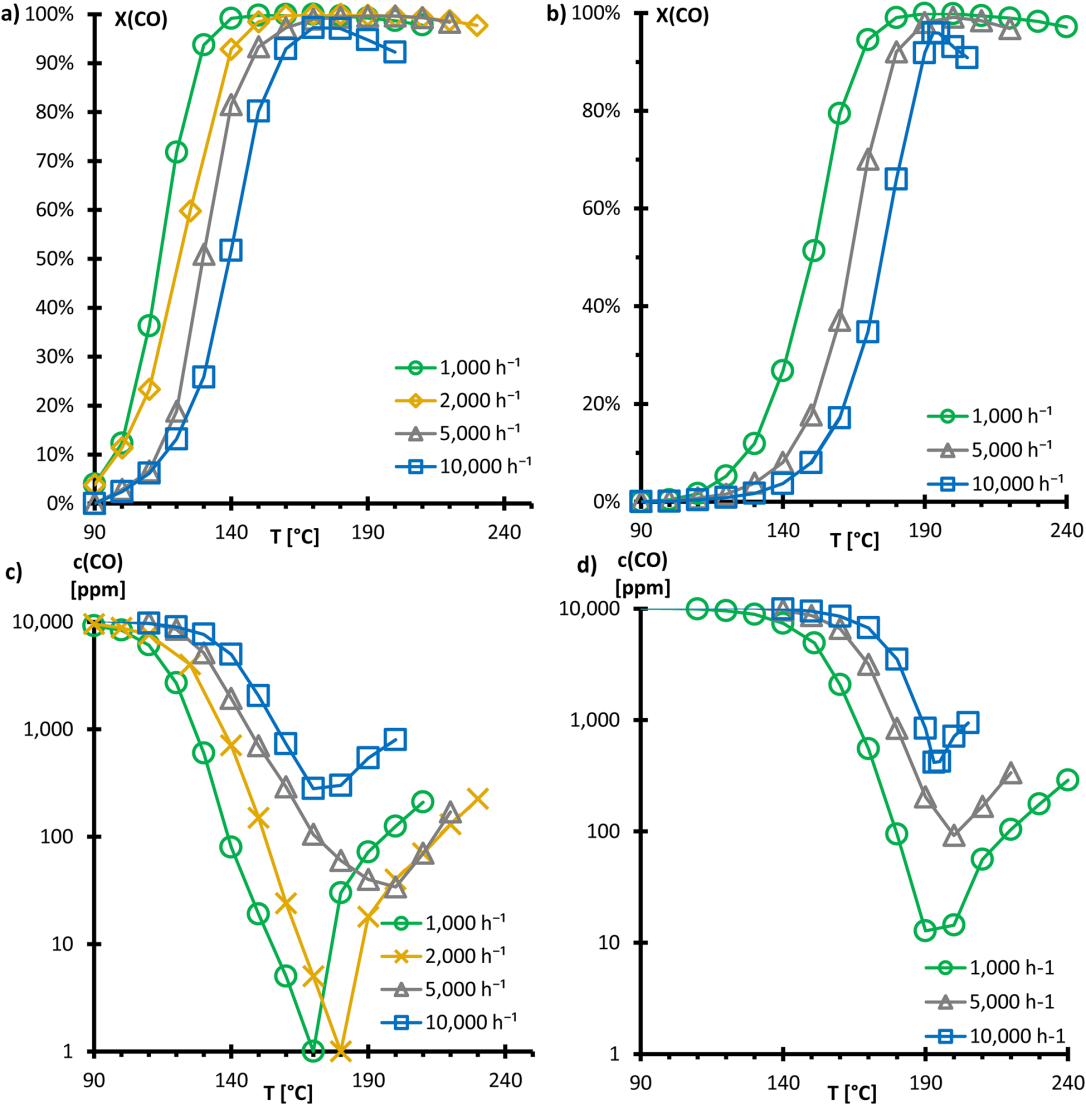
(**a**) CO conversion graphs, dry fuel gas (FG1) with Monolith #2 (CuO/CeO_2_), all λ = 2.5, varying GHSV. (**b**) CO conversion graphs, moist fuel gas (FG2) with Monolith #2 (CuO/CeO_2_), all λ = 2.5, varying GHSV. (**c**,**d**) corresponding CO concentrations at outlet.

Monolith #2, with a CuO–CeO_2_ coating of 768 mg, was used as synthesized without any pretreatment for the experiments in the next figure. Figure 6a shows CO conversion as a function of the reaction temperature measured with monolith #2. During these measurements, the GHSV was varied between 1000 h^−1^ and 10,000 h^−1^. The λ-value was 2.5 and in this case only the dry fuel gas 1 (FG1) was used. At a GHSV of 1000 h^−1^, CO conversion reached more than 99% in the temperature range between 140 and 210 °C with a slight decrease at 230 °C. In the case of a GHSV of 2000 h^−1^, the necessary temperature to reach CO conversion of more than 99% increased to 160 °C. CO conversion remained stable until 210 °C and again slightly decreased at 230 °C. For the GHSV of 5000 h^−1^, however, the temperature window with X(CO) > 99% became smaller and was between 170 and 210 °C. When further increasing the GHSV to 10,000 h^−1^, CO conversion did not exceed 99%, but a maximum of 97% was reached at temperatures of 170 °C and 180 °C. A sharp decline to approximately 92% was observed, when the temperature further raised to 200 °C. The corresponding CO concentrations at the outlet are depicted in Fig. 6c.

Figure 6b illustrates the CO conversion with the moist fuel gas (FG2) as a function of temperature using Monolith #2. The space velocity was varied from 1000 h^−1^ to 10,000 h^−1^. The λ-value was 2.50 for all conversions. At a GHSV of 1000 h^−1^, CO conversion of more than 99% was achieved in the temperature range of 180–220 °C. As the space velocity increased to 5000 h^−1^, CO conversion greater than 99% was achieved only at 200 °C. CO conversion higher than 98% was found in a temperature window from 190 to 210 °C. With a further increase to a GHSV of 10,000 h^−1^, a conversion maximum of only 96% was measured at 193 °C. At higher or lower temperatures, conversion strongly declined at this space velocity, with X(CO) = 66% at 180 °C and 91% at 205 °C. The corresponding CO concentrations at the outlet are depicted in Fig. 6d.

Supplementary Figure SI3 analyzes the influence of steam in the educt gas and compares the CO conversions with dry fuel gas (FG1) and moist fuel gas (FG2) using Monolith #2. Space velocities of 1000 h^−1^, 5000 h^−1^ and 10,000 h^–1^ were applied at a λ-value of 2.5. Common features in each case are that in the presence of steam in the fuel gas, the temperature interval of maximum CO conversion becomes smaller while the temperature level increases.

In more detail, it can be seen in Supplementary Fig. SI3 that at a GHSV of 1000 h^−1^, the start of the temperature window with more than 99% CO conversion being at 140 °C with FG1 is 40 K higher in the case of FG2 and only starts at 180 °C. Similar differences with respect to the CO conversion plots in the presence and absence of steam on Monolith #2 were also obtained for GHSVs of 5000 h^−1^ and 10,000 h^−1^. This inhibitory effect of water vapor on CO conversion when using CuO/CeO_2_ catalysts is well described in the literature^37–39^. It is assumed by Zou et al.^39^ that a competing adsorption of H_2_O at the catalytic sites and the formation of H_2_O–CO surface complexes causing the inhibition of the CO oxidation.

Supplementary Figure SI4 displays the corresponding conversion curves of the single-coated monolith #1 and the double-coated monolith #2. In all cases, it is clear that a higher CO conversion can always be achieved in a larger temperature window with the double-coated monolith, see also Table 1. The corresponding WHSV is indicated therein for comparison purposes.

**Table 1:** Comparing CO conversion for monoliths #1 and #2.

		Monolith #1	Monolith #2
5000 h^−1^ dry FG1	X(CO)	> 95%	> 99%
	T	155–160 °C	170–210 °C
	WHSV	88.8 L g^−1^ h^−1^	51.6 L g^−1^ h^−1^
10,000 h^−1^ dry FG1	X(CO)	> 86%	> 97%
	T	159–163 °C	170–180 °C
	WHSV	177.6 L g^−1^ h^−1^	103.1 L g^−1^ h^−1^
10,000 h^−1^ moist FG2	X(CO)	84%	> 91%
	T	191 °C	190–205 °C
	WHSV	177.6 L g^−1^ h^−1^	103.1 L g^−1^ h^−1^

Comparing the operational window of the single-coated monolith #1 with that of the double-coated monolith #2, it can be seen that the maximum CO conversion reaches a larger operational window with higher CO conversions at all operating points. The explanation for this difference is the higher loading of monolith #2 with catalytic material. Although the GHSV is identical for both monoliths, this results in a lower WHSV, which allows a larger temperature window for monolith #2 with higher CO conversions.

Snytnikov et al.^40^ have described conversions > 99.9% for a micro-reactor with catalytically coated stainless steel structures (5 wt% Cu/CeO_2−x_) with a reformate-like gas mixture, based on a WHSV of 240 L g^−1^ h^−1^ at 230 °C, exceeding the results given in this work.

**Figure 7:**
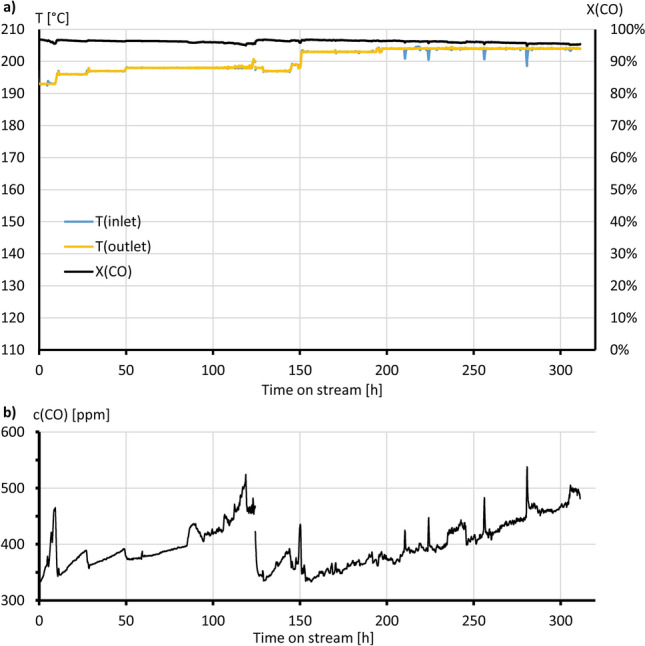
(**a**) CO conversion with Monolith #2, λ = 2.50; moist fuelgas (FG 2) at 10.000 h^−1^. (**b**) correspondig CO concentration at the outlet.

Furthermore, a long-term test was carried out with Monolith #2 over a period of 311 h (see Fig. 7). The moist educt mixture FG2 was used at a GHSV of 10,000 h^−1^ (WHSV = 103 L g^–1^ h^−1^) and a λ-value of 2.5. To ensure that always a maximum CO conversion was achieved the temperature window with maximum conversion was daily manually verified and the temperature in the reactor adjusted accordingly. The Fig. 7a shows the temperature at the inlet and outlet of the monolith, as well as the CO conversion. The temperature was kept constant at the inlet and outlet of the coated monolith. In the diagram, only minor short-term variations of these temperatures can be seen, which did not induce any significant effect on the CO conversion. Figure 7b depicts additionally the CO product concentration.

Over the course of the 311 h, the temperature at which the maximum conversion could be achieved rose from an initial 193 °C by 10 K, to 203 °C. Thereby, the maximum conversion decreased by 1.4 percentage points from X(CO) = 96.8% to X(CO) = 95.4%. This means that at the beginning of the long-term test, there were about 350 ppm of carbon monoxide in the product gas. This value increased up to 500 ppm after 311 h. In comparison, Maeda et al.^41^ described a water-tolerant monolithic 4 wt% Pt-0.5wt% Fe/modernite catalyst. At a space velocity of 9500 h^−1^ and a λ-value of 2.0, it was possible to maintain a CO conversion of more than 99% at 130 °C for 200 h.

## Conclusions

The direct coating of a ceramic monolith made of cordierite using the urea-combustion method is a new and promising approach that has not yet been described in the literature. Already in a single coating step, the surface of a ceramic monolith can be coated with a 20 times higher amount of catalyst, which also promises very good adhesion. A second coating of the monolith improves the catalytic activity substantially. Compared with a large number of other publications on CO-PrOx catalysts on monolithic supports (cf. Supplementary Table SI1 Monoliths overview), the results of applying a double direct coating using the urea-combustion method are promising, even if the long-term stability of the catalyst in the presence of water in the fuel gas still displays potential for improvement.

The CO conversion in the long-term experiment would still be not high enough for a single-stage PrOx reactor to fall below a CO concentration of 100 ppm. However, the design temperature of 200–300 °C with CO conversions close to 100%, as described in the introduction, was achieved satisfactorily. In order for operation of the fuel processing system for a PEFC, the outlet temperature of the WGS and the inlet of the sub-sequent PrOx-reactor must still be fine-tuned to each other. A comparison of the long-term stability of different monolithic catalysts over a few hundred hours is not possible, as hardly any studies have been published.

## Supplementary Information

Supplementary Information 1.

Supplementary Information 2.

## Data Availability

All data generated or analyzed during this study are included in this article, its supplementary information or are available from the corresponding author on reasonable request.

## Abbreviations

EDX: Energy-dispersive X-ray spectroscopy
EPR: Electron paramagnetic resonance spectroscopy
FG: Fuel gas
FTIR: Fourier-transform infrared spectroscopy
GHSV: Gas hourly space velocity
PE(M)FC: Polymer electrolyte (membrane) fuel cell
PrOx: Preferential oxidation
SEM: Scanning electron microscopy
WGS: Water-gas shift
XRD: X-ray diffraction
$\dot{\mathrm{n}}$: Molar flow [mol/s]
X: Conversion [%]
λ: Air–fuel ratio [–]
Θ: Angle [°]
