# Supplementary material for: An improved preparation method for a CuO/CeO2-coated monolith for the CO–PrOx reaction
Source: Sci Rep. 2023 Jun 8;13:9345. doi: 10.1038/s41598-023-36423-7 (PMC10250403; doi:10.1038/s41598-023-36423-7)
Supplement: Supplementary file 2 — Supplementary Information 2. [file 41598_2023_36423_MOESM2_ESM.pdf]

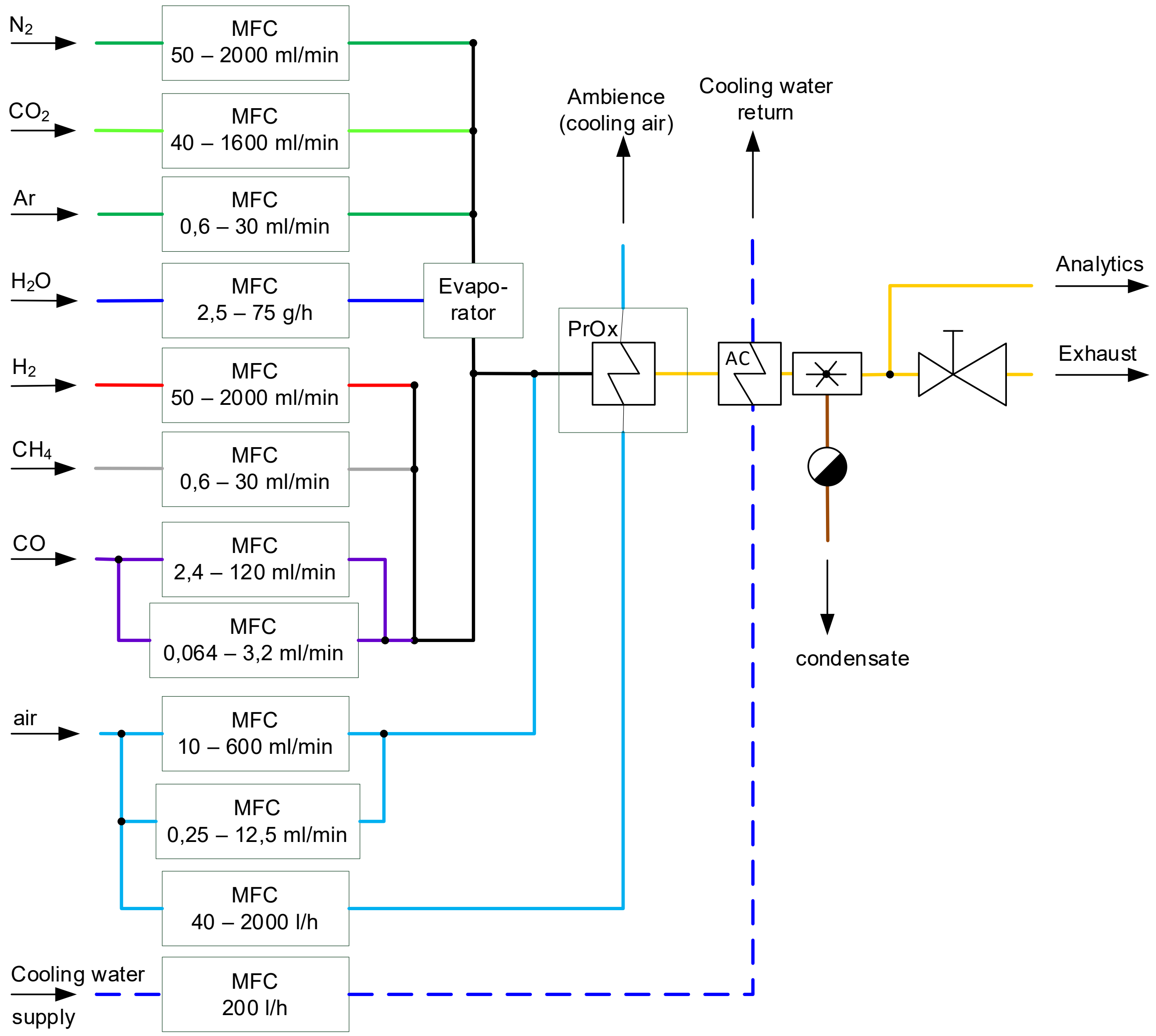

Figure SI1. Simplified block diagram of the test rig

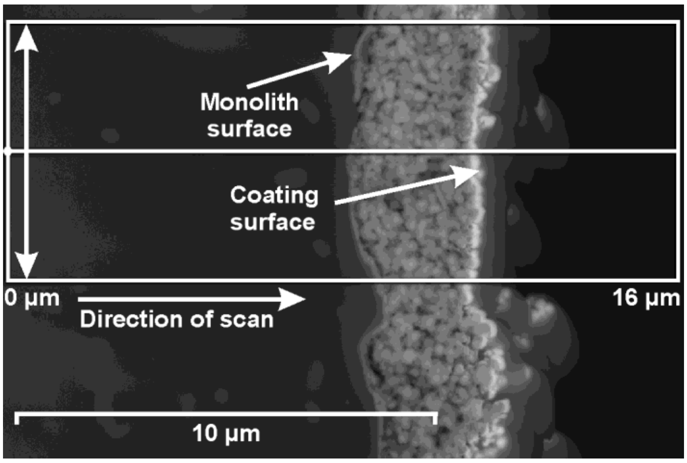

Figure SI2. Overview image of the EDX line scan

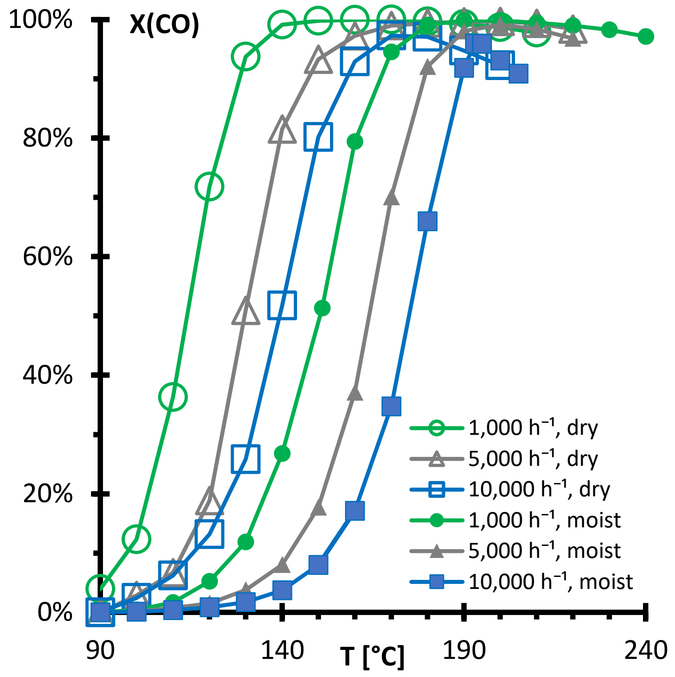

Figure SI3. CO conversion graphs, dry fuel gas (FG1) & moist fuel gas (FG2) with Monolith #2 (CuO/CeO_2), all λ = 2.5, varying GHSV.

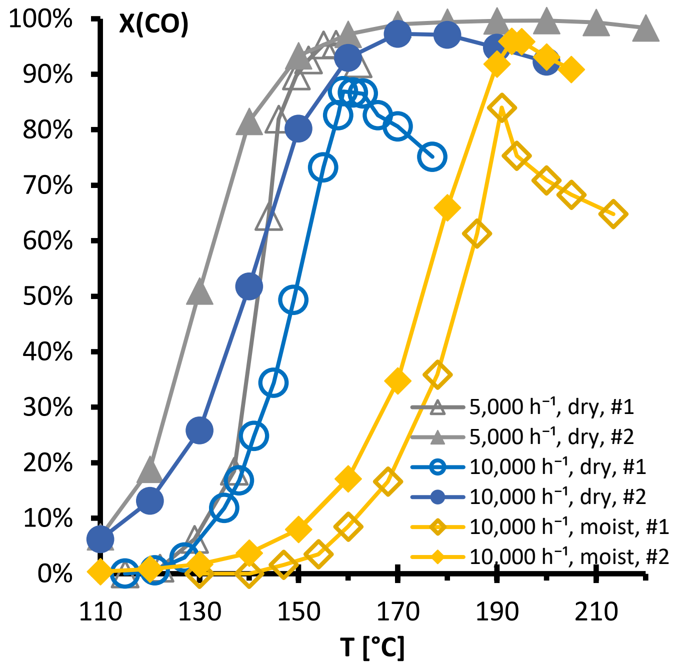

Figure SI4. CO conversion graphs, comparing single coated monolith #1 and double coated monolith #2 (CuO/CeO_2), all λ = 2.5, varying GHSV.

Table SL1. Monoliths overview

composition	Washcoat carrier	preparation/ coating method	layer [µm]	pore size [nm]	coating quantity [mg/cm³]	support	l [mm]	D [mm]	V [cm³]	H_2	CO_2	H_2O	CO	N_2	O_2	GHSV [h^{-1}]	λ	T [°C]	X(CO) [%]	S(CO) [%]	X(O_2) [%]	Y(CO) [%]	remark	ref.
						monolith				Feed [mol.%]														
5 wt.% Pt, 0.5 wt.% Fe	Al_2O_3	dip-coating	15 - 20	-	Al_2O_3: 98<br>Pt: 4.9<br>Fe: 0.49	honeycomb ceramic<br>400 cpsi	50,8	25,4	25.7	42.0	9.0	12.0	1	35 - 35.75	0.25 - 1.0	10000	2.0	100<br>at inlet	47	40	100	18,8		[1]
unknown, propietary<br>Engelhard Selectra-PROX I	-	-	-	-	-	honeycomb<br>cordierite, 600 cpsi	127	76,2	579	38.4	19.9	16.8	0.2	24.7		18000	1.35			69	100			[2]
0.5 wt.% Pt	Al_2O_3	-	-	-	-	cordierite<br>600 cpsi	-	-	-	30.0	17.0	15.0	0.003 - 1.0	balance	0.003 - 1.0	-	-	-	-	-	-	-		[3]
0.5 wt.% Pt																10000		170	99	50	100	50		
																30000		180	95	61	100	58		
0.5 wt.% Pd	γ-Al_2O_3	impregnation	-	-	-	honeycomb cordierite<br>400 cpsi	-	-	-	40.0	-	20.0	0.8	38.4	0,8	30000	2.0	240-280	25	12	100	3		[4]
0.5 wt.% Rh																30000		120-150	68	35	100	24		
0.5 wt.% Ru																30000		130	52	27	100	14		
0.1 wt.% Pt	12 % γ-Al_2O_3	dip-coating<br>slurry-sol-slurry	-	-	-	honeycomb metallic<br>Fe-20Cr-5Al<br>400 cpsi	-	-	-	39.6	19.8	16.5	1.0	22.1	1.0	10000	2.0	198-206	96	47,5	-	46		[5]
4 wt.% Pt, 0.5 wt.% Fe,<br>Mordenite	-	dip-coating	14		100	honeycomb<br>cordierite, 400 cpsi	15	12	1.70	63.0	20.0	15.0	1.0	-	1.0	9500	2.0	100-150	99,95	50	100	50,0		[6]
15 mol.% Cu, 85 mol.% Ce			-	-	-													165-185	100	70	100	70,0		
15 mol.% Cu, 8.5 mol.% Nd,<br>76.5 mol.% Ce	Al_2O_3	dip-coating	-	-	-	honeycomb metallic<br>Fe-20Cr-5Al<br>400 cpsi	15	7	0.577	50.0	7.5	10.0	0.5	31.5	0.5		2.0	165-205	100	70		70,0		[7]
15 mol.% Cu, 8.5 mol.% Zr,<br>76.5 mol.% Ce			-	-	-													165-205	100	75		75,0		
4 wt.% Pt, 0.5 wt.% Fe,<br>Mordenite	-	dip-coating<br>alumina-sol	-	-	100	honeycomb<br>cordierite, 400 cpsi	15	12	1.70	73.0	20.0	5.0	1.0	-	1.0	9500	2.0	130	99.9	-	-	0.0	after 200 h<br>H_2O-tolerance<br>durability test	[8]
		dip-coating<br>silica-sol			100	honeycomb<br>cordierite, 400 cpsi												100	99.8	-	-	0.0		
2 wt.% Pt, Z-PM zeolith	-	impregnation,<br>dip-coating	50 - 75	-	50	honeycomb<br>cordierite, 225 cpsi	-	-	-	97.5	-	-	1.0	-	1.5	225000	3.0	100-150	65	>50	-	-	coating quantity<br>approximated	[9]
2 wt.% Pt,SiO_2																		250	82	52	-	-		
1 wt.% Pt	γ-Al_2O_3	impregnation	-	3.8	-	macro-porous<br>Al_2O_3	-	-	-	50.0	-	-	1.0	48.0	1.0	20000	2.0	225-250	99.6	42	100	41.8		[10]
1 wt.% Pt, 2 wt.% K	γ-Al_2O_3		-	3.0	-													200-250	>99	42	100	41.6	molar ratio K/Pt = 10	
1 wt.% Pt, 1.5 wt.% Ni	γ-Al_2O_3	impregnation	-	-	-	macro-porous<br>Al_2O_3	-	-	-	50.0	12.5	15.0	1.0	20.5	1.0	16000	2.0	140-180	>99	51	-	51		[11]
CuO-CeO_2 (6.8 wt.% Cu)	-	impregnation	9	4.5	-	honeycomb<br>cordierite, 400 cpsi	21	9	1.34	42.0	15.0	15.0	0.7	He 26.6	0.7	9000	2.0	135-158	100	-	-	-	Cu/Ce-ratio=0.13	[12]
CuO-CeO_2 (8.6 wt.% Cu)			12	4.4	-													128-144	100	-	-	-	Cu/Ce-ratio=0.17	
M1 Co-ZrO_2 (15.9 wt.% Co)			-	-	41													250-260	70	-	-	-	slurry ZrO_2, 10wt.%/HAc	
M2 Co-ZrO_2 (12.1 wt.% Co)			-	-	35													-	-	-	-	-	slurry ZrO_2, 10wt.%/H_2O	
M3 Co-ZrO_2 (11.1 wt.% Co)			-	-	40													250-260	85	-	-	-	slurry ZrO_2, 20wt.%/HAc	
M3* Co-ZrO_2 (21.5 wt.% Co)			-	-	38													240	90	-	-	-	slurry ZrO_2, 20wt.%/HAc	
M4 Co-ZrO_2 (10.6 wt.% Co)	ZrO_2	impregnation	-	-	76	honeycomb<br>cordierite, 400 cpsi	20	10x10	2.00	40.0	-	-	1.0	He 58.0	1.0	-	2.0	220-250	>90	-	-	-	slurry NYACOL(ZrO_2),<br>stability test 100h	[13]
M5 Co-ZrO_2 (10.6 wt.% Co)			-	-	55													240-260	85	-	-	-	slurry ZrO_2,<br>20wt.%/NYACOL(ZrO_2)	
M6 Co-ZrO_2 (6.9 wt.% Co)			-	-	66													-	-	-	-	-	slurry NYACOL(Al_2O_3)-<br>ZrO_2 20 wt.%/HAc	
C1 Co-CeO_2 (11.2 wt.% Co)			-	-	23													200-210	98	-	-	-		
C2 Co-CeO_2 (10.1 wt.% Co)	CeO_2	impregnation	-	-	53	honeycomb<br>cordierite, 400 cpsi	20	10x10	2.00	40.0	-	-	1.0	He 58.0	1.0	-	2.0	200-210	95	-	-	-	coating quantity<br>calculated	[14]
C3 Co-CeO_2 (10.0 wt.% Co)			-	-	23													200-210	98	-	-	-		
0.02 wt.% Pt, 4.5 wt.% CuO	CeO_2	SFRD	-	-	-		-	-	-	41.0	-	-	0.5	58.0	0.5	-	2.0	150	>90	-	-	-		[15]

composition	Washcoat carrier	preparation/ coating method	layer [µm]	pore size [nm]	coating quantity [mg/cm³]	monolith support	monolith l [mm]	monolith D [mm]	monolith V [cm³]	Feed [mol.%] H_2	Feed [mol.%] CO_2	Feed [mol.%] H_2O	Feed [mol.%] CO	Feed [mol.%] N_2	Feed [mol.%] O_2	GHSV [h^{-1}]	λ	T [°C]	X(CO) [%]	S(CO) [%]	X(O_2) [%]	Y(CO) [%]	remark	ref.
0.01 wt.% Pt, 2.0 wt.% CuO	CeO_2	SFRD				α-Al_2O_3-foam, 20 ppi												140-150	>90	-	-	-	SFRD supercritical fluid reactive deposition	
0.03 wt.% Pt, 5.8 wt.% CuO	CeO_2	SFRD																90-150	<60	-	-	-		
1.0 wt.% Au, 1.25 wt.% MgO	Al_2O_3	homogenous deposition-precipitation (urea)	-	-	-	honeycomb cordierite	20	11	1.90	61.7	-	-	1.0	He 36.3	1.0	-	3.2	90	60	-	-	-		[16]
Pt-promoted CuFe-catalyst*	Al_2O_3	impregnation	-	-	-	honeycomb cordierite, 400 cpsi	76.2	19.1	21.7	45.7	17.5	25.0	0.222	11.4	0.222	24000	2.0	70-150	>96	>50	-	>48	propietary	[17]
CuO_x-CeO_2 (14 mol.% Cu, 86 mol.% Ce)	enamel	coprecipitation	-	8	103	enameled steel monolith	30	17	6.81	50.0	-	-	1.0	48.0	1.0	7000	2.0	170-200	>99	-	-	-		[18]
CuO_x-CeO_2 (4.2 wt.% CuO)	-	wet impregnation, dip-coating	12-15	-	230	honeycomb cordierite, 900 cpsi	12	16	2.41	50.0	-	-	0.5	48.6	0.9	-	3.6	90-120	>95	30-60	-	30-60		[19]
											15	10		23.6				145	>90	<30	-	<30		
2 wt.% Au/TiO_2		incipient wetness impregnation			23.0	anodized Al- monolith	15	14	2.31	composition not given						1040	3.0	80-110	50	<20	-	<10		[20]
CuO_x-CeO_2 (4.2 wt.% CuO), S1-M		wet impregnation, dip-coating	15			honeycomb cordierite, 400 cpsi	12	16	2.41	50.0	-	-	0.5	23.6	0.9		3.6	140-200	>99	28-62	-	28-80		
CuO_x-CeO_2 (4.2 wt.% CuO), S2-M		wet impregnation, dip-coating	15			honeycomb cordierite, 400 cpsi	12	16	2.41	50.0	-	-	0.5	23.6	0.9		3.6	160-180	>99	33-48	-	28-80		
CuO_x-CeO_2 (4.2 wt.% CuO), S3-M		wet impregnation, dip-coating	15			honeycomb cordierite, 400 cpsi	12	16	2.41	50.0	-	-	0.5	23.6	0.9		3.6	140-200	>99	28-78	-	28-80		[21]
CuO_x-CeO_2 (4.2 wt.% CuO), S5-M		wet impregnation, dip-coating	15			honeycomb cordierite, 400 cpsi	12	16	2.41	50.0	-	-	0.5	23.6	0.9		3.6	130-200	>99	28-87	-	28-80		
CuO_x-CeO_2 (4.2 wt.% CuO), S6-M		wet impregnation, dip-coating	15			honeycomb cordierite, 400 cpsi	12	16	2.41	50.0	-	-	0.5	23.6	0.9		3.6	200	98	31	-	28-80		
CuO (4.8 wt.% Cu)	CeO_2	wet impregnation, dip-coating			28.9	3d-printed resin	10	15	1.77	30.0	-	-	2.0	He 66.0	2.0	2000	2.0	135	95	90	-	86	10 h experiment	[22]
CuO-CeO_2 /celite (CuO 4.6 wt.%, CeO_2 18.7 wt.%, Cu/Ce=0.55)		co-impregnation, dip-coating	20-30		46													150-185	>98					
CuO-CeO_2 /aerosil (CuO 4.9 wt.%, CeO_2 18.9 wt.%, Cu/Ce=0.55)		co-impregnation, dip-coating	20-30		47	honeycomb cordierite, 400 cpsi	10	10x10	1.0									185	91					[23]
CuO-CeO_2 /SBA-15 (CuO 4.9 wt.%, CeO_2 19.1 wt.%, Cu/Ce=0.55)		co-impregnation, dip-coating	20-30		48													185	85					

References Supplementary Table1 Monoliths overview

[1] G.W. Roberts, P. Chin, X. Sun, J.J. Spivey, Preferential oxidation of carbon monoxide with Pt/Fe monolithic catalysts: Interactions between external transport and the reverse water-gas-shift reaction, Appl. Catal., B, 46 (2003) 601-611. http://dx.doi.org/10.1016/j.apcatb.2003.07.002.

[2] R.K. Ahluwalia, Q. Zhang, D.J. Chmielewski, K.C. Lauzze, M.A. Inbody, Performance of CO preferential oxidation reactor with noble-metal catalyst coated on ceramic monolith for on-board fuel processing applications, Catal. Today, 99 (2005) 271-283. http://dx.doi.org/10.1016/j.cattod.2004.10.015.

[3] E.J. Bissett, S.H. Oh, R.M. Sinkevitch, Pt surface kinetics for a PrOx reactor for fuel cell feedstream processing, Chem. Eng. Sci., 60 (2005) 4709-4721. http://dx.doi.org/10.1016/j.ces.2005.02.069.

[4] S. Zhou, Z. Yuan, S. Wang, Selective CO oxidation with real methanol reformate over monolithic Pt group catalysts: PEMFC applications, Int. J. Hydrog. Energy, 31 (2006) 924-933. http://dx.doi.org/10.1016/j.ijhydene.2005.07.014.

[5] J. Zhang, J. Jia, C. Zhang, S. Wang, Preparation of Pt/Al_2O_3 Metallic Honeycomb Catalyst and Its Catalytic Properties for CO Preferential Oxidation, Chin. J. Catal., 29 (2008) 421-425. https://www.cjcatal.com/EN/Y2008/V29/I5/421.

[6] N. Maeda, T. Matsushima, H. Uchida, H. Yamashita, M. Watanabe, Performance of Pt-Fe/mordenite monolithic catalysts for preferential oxidation of carbon monoxide in a reformate gas for PEFCs, Appl. Catal., A, 341 (2008) 93-97. http://dx.doi.org/10.1016/j.apcata.2008.02.022.

[7] S.H. Zeng, Y. Liu, Nd- or Zr-modified CuO-CeO_2 /Al_2O_3 /FeCrAl monolithic catalysts for preferential oxidation of carbon monoxide in hydrogen-rich gases, Appl. Surf. Sci., 254 (2008) 4879-4885. http://dx.doi.org/10.1016/j.apsusc.2008.01.168.

[8] N. Maeda, T. Matsushima, M. Kotobuki, T. Miyao, H. Uchida, H. Yamashita, M. Watanabe, H_2O-tolerant monolithic catalysts for preferential oxidation of carbon monoxide in the presence of hydrogen, Appl. Catal., A, 370 (2009) 50-53. http://dx.doi.org/10.1016/j.apcata.2009.09.010.

[9] G. Neri, G. Rizzo, F. Corigliano, I. Arrigo, M. Caprì, L. De Luca, V. Modafferi, A. Donato, A novel Pt/zeolite-based honeycomb catalyst for selective CO oxidation in a H_2-rich mixture, Catal. Today, 147 (2009) S210-S214. http://dx.doi.org/10.1016/j.cattod.2009.07.049.

[10] Y. Zhang, C.Y. Zhao, H. Liang, Y. Liu, Macroporous monolithic Pt/γ-Al_2O_3 and K-Pt/γ-Al_2O_3 catalysts used for preferential oxidation of CO, Catal. Lett., 127 (2009) 339-347. http://dx.doi.org/10.1007/s10562-008-9686-z.

[11] S. Lu, Y. Liu, Y. Wang, Meso-macro-porous monolithic Pt-Ni/Al_2O_3 catalysts used for miniaturizing preferential carbon monoxide oxidation reactor, Chem. Commun., 46 (2010) 634-636. http://dx.doi.org/10.1039/b912769k.

[12] J.L. Ayastuy, N.K. Gamboa, M.P. González-Marcos, M.A. Gutiérrez-Ortiz, CuO/CeO_2 washcoated ceramic monoliths for CO-PROX reaction, Chem. Eng. J., 171 (2011) 224-231. http://dx.doi.org/10.1016/j.cej.2011.03.006.

[13] L.E. Gómez, I.S. Tiscornia, A.V. Boix, E.E. Miró, Co/ZrO_2 catalysts coated on cordierite monoliths for CO preferential oxidation, Appl. Catal., A, 401 (2011) 124-133. http://dx.doi.org/10.1016/j.apcata.2011.05.007.

[14] L.E. Gómez, I.S. Tiscornia, A.V. Boix, E.E. Miró, CO preferential oxidation on cordierite monoliths coated with Co/CeO_2 catalysts, Int. J. Hydrog. Energy, 37 (2012) 14812-14819. http://dx.doi.org/10.1016/j.ijhydene.2012.01.159.

[15] S. Lang, M. Türk, B. Kraushaar-Czarnetzki, Novel PtCuO/CeO_2/α-Al_2O_3 sponge catalysts for the preferential oxidation of CO (PROX) prepared by means of supercritical fluid reactive deposition (SFRD), J. Catal., 286 (2012) 78-87. http://dx.doi.org/10.1016/j.jcat.2011.10.017.

[16] S. Özdemir, Z.I. Önsan, R. Yildirim, Selective CO oxidation over monolithic Au-MgO/Al_2O_3 catalysts, J. Chem. Technol. Biotechnol., 87 (2012) 58-64. http://dx.doi.org/10.1002/jctb.2682.

[17] Q. Zhang, L. Shore, R.J. Farrauto, Selective CO oxidation over a commercial PROX monolith catalyst for hydrogen fuel cell applications, Int. J. Hydrog. Energy, 37 (2012) 10874-10880. http://dx.doi.org/10.1016/j.ijhydene.2012.04.032.

[18] M.I. Domínguez, A. Pérez, M.A. Centeno, J.A. Odriozola, Metallic structured catalysts: Influence of the substrate on the catalytic activity, Appl. Catal., A, 478 (2014) 45-57. http://dx.doi.org/10.1016/j.apcata.2014.03.028.

[19] P.S. Barbato, A. Di Benedetto, G. Landi, L. Lisi, CuO/CeO_2 based monoliths for CO preferential oxidation in H_2-rich streams, Chem. Eng. J., 279 (2015) 983-993. https://dx.doi.org/10.1016/j.cej.2015.05.079.
[20] E. Adrover, D. Boldrini, N.J. Divins, A. Casanovas, G. Tonetto, E. López, J. Llorca, Study of Cu-Zn and Au/TiO_2 catalysts on anodized aluminum monoliths for hydrogen generation and purification, Int. J. Chem. Reactor Eng., 14 (2016) 831-842. http://dx.doi.org/10.1515/ijcre-2015-0119.
[21] G. Landi, Barbato, Paola Sabrina, A. Di Benedetto, L. Lisi, Optimization of the preparation method of CuO/CeO_2 structured catalytic monolith for CO preferential oxidation in H_2-rich streams, Appl. Catal., B, 181 (2016) 727-737. https://dx.doi.org/10.1016/j.apcatb.2015.08.040.
[22] C.Y. Chaparro-Garnica, A. Davó-Quiñonero, E. Bailón-García, D. Lozano-Castelló, A. Bueno-López, Design of Monolithic Supports by 3D Printing for Its Application in the Preferential Oxidation of CO (CO-PrOx), ACS Applied Materials & Interfaces, 11 (2019) 36763-36773. http://dx.doi.org/10.1021/acsami.9b12731.
[23] I.S. Tiscornia, A.M. Lacoste, L.E. Gómez, A.V. Boix, $CuO–CeO_2/SiO_2$ coating on ceramic monolith: Effect of the nature of the catalyst support on CO preferential oxidation in a H_2-rich stream, Int. J. Hydrog. Energy, 45 (2020) 6636-6650. http://dx.doi.org/10.1016/j.ijhydene.2019.12.126.
